# Evaluation of cerebral dysfunction in patients with chronic kidney disease using neuropsychometric and neurophysiological tests

**DOI:** 10.1080/0886022X.2021.1901740

**Published:** 2021-03-24

**Authors:** Fabiola Sanchez-Meza, Aldo Torre, Lilia Castillo-Martinez, Sofia Sanchez-Roman, Luis Eduardo Morales-Buenrostro

**Affiliations:** a Department of Nephrology and Mineral Metabolism, Instituto Nacional de Ciencias Médicas y Nutrición “Salvador Zubirán”, Tlalpan, Mexico; b Liver Unit, Department of Gastroenterology, Instituto Nacional de Ciencias Médicas y Nutrición “Salvador Zubirán”, Tlalpan, Mexico; c Department of Clinical Nutrition, Instituto Nacional de Ciencias Médicas y Nutrición “Salvador Zubirán”, Tlalpan, Mexico; d Neurology and Psychiatry Department, Instituto Nacional de Ciencias Médicas y Nutrición “Salvador Zubirán”, Tlalpan, Mexico

**Keywords:** Critical flicker frequency, chronic kidney failure, uremic encephalopathy, cerebral dysfunction, uremia

## Abstract

**Background:**

Uremic encephalopathy is defined as cerebral dysfunction due to toxin accumulation in patients with chronic kidney disease (CKD). This condition is characterized by subtle to florid symptoms, and its clinical course is always progressive when untreated but partially reversible with renal replacement therapy. While no test exists to measure subclinical uremic encephalopathy, two tests have been validated to measure minimal hepatic encephalopathy: the critical flicker frequency (CFF) test and the psychometric hepatic encephalopathy score (PHES).

**Objective:**

To use CFF and PHES to measure the prevalence of cerebral dysfunction in individuals with CKD.

**Methods:**

This cross-sectional study included a total of 69 patients with stage-5 CKD. Cutoff points for minimal encephalopathy were established using existing clinical guidelines: ≤39 Hz for CFF and < −4 for PHES. All participants were also screened for cognitive function and depression.

**Results:**

Eighteen cases (26.1%) of cerebral dysfunction linked to uremic encephalopathy were detected with CFF, while twelve (17.4%) were detected by PHES; only six cases (8.7%) were diagnosed by both methods. Half of the cases (50%) had diabetes, and 61% were on hemodialysis. Cognitive function scores did not differ significantly between those receiving dialysis, hemodialysis, or no renal replacement therapy.

**Conclusions:**

It is essential to identify cerebral dysfunction when uremic encephalopathy is in early subclinical stages to reduce preventable events as traffic and work accidents.

## Introduction

Chronic kidney disease (CKD) is a public health problem and the worldwide prevalence is estimated at 10% [1]. The uremic state in CDK patients affect several organ and system functions including neurological system with development of uremic encephalopathy (UE), that is associated with the rapidity of accumulation of uremic toxins or renal replacement therapy quality [2–4]. The pathophysiology of UE is complex and unclear; range of symptoms goes from fatigue, irritability, impaired cognition, perceptual errors to tonic-clonic seizures and progression to coma [5–7], these symptoms of uremia are improved by dialysis.

There is no specific confirmatory test to diagnose UE. Evaluation of UE includes laboratory, neurological and neuroimaging techniques. For neurological evaluation, the electroencephalogram (EEG) is non-diagnostic and its findings are frequently nonspecific. Recommended cognitive tests are the trail-making test, term memory tests, and choice reaction time tests [3].

On the other hand, it is important to identify the UE present in a pre-clinical stage, known as mild, latent, asymptomatic, subclinical or minimal; characterized by poor concentration, forgetfulness and personality changes [6,8], frequently only perceived by family member or caregiver. The diagnosis of subclinical uremic encephalopathy is not a priority in the daily practice, but this condition affects the quality of life of patients and caregivers and could be associated with traffic or work accidents, due to reduction of driving or occupational skills and cognitive impairment [9,10].

No studies exist on the prevalence of cerebral dysfunction linked to subclinical UE in individuals with kidney disease. Minimal hepatic encephalopathy (mHE) occurs in 30–85% of patients with liver cirrhosis [11–13] and has been associated with a higher risk of car accidents, impact on the quality of life, and legal implications [10,14]. Two tools have been validated for diagnosing mHE: the Psychometric Hepatic Encephalopathy Score (PHES) [15,16] the Critical flicker frequency (CFF) [17–19]. These neurophysiological tests are simple, rapid, effective, and reliable, with high specificity and reproducibility and can predict the risk for mortality and developing overt encephalopathy [20–23].

Both the PHES and CFF methods have been validated in Mexican patients with CHE (Román-Calleja B, *et al.* 2017, unpublished data). As minimal encephalopathy symptoms are similar for both cases (uremic and hepatic), we propose to use the same instruments that have been demonstrated to be useful for detecting mHE. Therefore, this study aimed to identify the prevalence of cerebral dysfunction in a group of CKD patients using PHES and CFF. As a secondary objective, we tested a subgroup of those patients before and after hemodialysis to evaluate the change in PHES and CFF.

## Methods

### Study design and recruitment

This cross-sectional study was conducted in 69 patients with stage 5 CDK in the years 2015 and 2016. We recruited patients between the ages of 18 to 60 years from Department of Nephrology and Mineral Metabolism at *Instituto Nacional de Ciencias Médicas y Nutrición “Salvador Zubirán” (INCMNSZ).* Patients were included in the study if they were diagnosed with stage 5 CKD and had the intact motor functions necessary to complete neuropsychologic assessments. Any patients with diabetes and retinopathy were only included if they had mild retinopathy that did not compromise their performance.

Patients with color blindness or mature cataracts or those who used antileptic drugs or antidepressants were excluded. Patients were also excluded if they had psychiatric or neurological disorders. All participants signed an informed consent form.

All clinical and research activities were carried out in accordance with the Declaration of Helsinki. The study was approved by the Institutional Ethics and Human Research Committees (Ref. 1525). All data were collected through face-to-face interviews conducted by standardized coauthors; laboratory data were obtained from medical records.

### Cerebral dysfunction

The cerebral dysfunction evaluation was based on two tests, CFF and PHES. The PHES is currently internationally recommended as the gold standard for diagnosing minimal hepatic encephalopathy (mHE) and is validated in Spanish, Italian, German, and Mexican languages. The PHES is adjusted to educational level and age in each national population.

PHES assesses several neurological functions, such as motor speed, attention, concentration, visual perception, visual-spatial orientation, memory, and visual construction. It is composed of five dominium: the number connection test-A (NCT-A), number connection test-B (NCT-B), serial dotting test (SDT), line tracing test (LTT), and digit symbol test (DST). The PHES is performed in a quiet room with a fixed table, adequate lighting, and medium point pen. The individual dominium test score was summarized to a sum score, with scores ranging from +6 to −18 points. Patients were classified as having cerebral dysfunction when the PHES test score was below −4[16].

Critical flicker frequency (CFF) is a neurophysiological technique that measures the capability to detect flickering light directly influenced by cortical activity in the central nervous system [21,24]. We used a Hepatonorm Analyzer (nevoLAB GmbH, Maierhöfen, Germany). Participants first learned about the test and were taught how to take it; all participants first took part in a practice test to familiarize themselves with the apparatus. In the official test, the CFF frequencies were measured nine times, and the mean value was recorded. The device starts the flickering light at 60 Hz, gradually reduces the frequency by 0.1 Hz per second, and patients indicate when they first identify the light and the moment that it starts to blink [17]. Patients were classified as having subclinical UE when the flicker score was below the cutoff value of 39 Hz [11,25,26]. Patients with mild diabetic retinopathy had their capacity to identify the red point and movement verified before the test [27].

### Assessment of cognitive function and depression

We assessed global cognitive function with the Mini-Mental State Examination (MMSE); this test takes approximately seven minutes to complete and includes concentration, orientation, language, praxis, and memory components [28–30]. The maximum score on the MMSE is 30 points; scores < 24 suggest the presence of cognitive impairment decline: 23–21, mild decline; between 20 and 11, moderate decline; and <10, severe decline.

The Beck Depression Inventory (BDI-II) score was used to measure the intensity of depression. The BDI-II uses a 21-item scale with a maximal score of 63 points; scores ranging from 0 to 13 denote a normal score; 14–19, mild depression; 20–28, moderate depression; and 29–63 severe depression [31,32]. Patients with depression were referred to the Institutional Psychology Department.

### Statistical analysis

Data were first summarized through descriptive measures; categorical variables were presented as frequencies and proportions. All quantitative variables were analyzed for normal distribution with a Kolmogorov–Smirnov Z test. Data were then summarized according to whether they fulfilled parametric assumptions or not: continuous normal data were summarized with mean ± standard deviation, while continuous non-normal data were summarized with median and interquartile range.

For analysis, quantitative normal data were evaluated through a Student *t*-test, and quantitative non-normal data were evaluated with a Mann–Whitney U test. A Chi [2] was used to compare categorical variables. A Fisher’s exact test was used for two sub-analyses comparing the frequency of cerebral dysfunction in participants with and without cognitive impairment, and the frequency of cerebral dysfunction in diabetic participants with and without retinopathy. We used paired *t*-tests to compare PHES and CFF values before and after hemodialysis. All data were analyzed using the Statistical Package for the Social Sciences Version 21.0. (IBM SPSS Statistics for Windows, IBM Corp, Armonk, NY). A *p* value <0.05 was considered statistically significant.

## Results

A total of 69 participants were included in the study; their baseline clinical and demographic characteristics are shown in Table 1. Participants had been diagnosed with CKD an average of 1.5 years prior to the study, with diabetes being the predominant etiology in 50% of the patients and 61% of the patients being on hemodialysis.

**Table 1.** Baseline characteristics of the study population.

Variables	Group 1 CKD *n* = 69 (%)
Age, years	35 [26–48]
18–28	26 (38)
29–38	15 (22)
39–48	11 (16)
49–58	11 (16)
>59	6 (8)
Male, Gender	38 (55)
Time with CKD, years	1.5 [0.6 − 2.6]
CKD etiology	
Diabetes mellitus	39 (50)
Unknown	21 (37)
Secondary glomerulonephritis	2 (3)
Primary glomerulonephritis	1 (1)
Hypertension	6 (9)
Dialysis treatment	
Peritoneal dialysis	20 (29)
Hemodialysis	42 (61)
Pre-dialysis	7 (10)
Hemoglobin (g/L)	10.6 ± 1.9
Serum albumin (g/L)	4.2 ± 0.6
Serum creatinine (mg/dl)	10.5 ± 4.7
Blood urea nitrogen (mg/dl)	62.0 ± 20.4
Glucose levels (mg/dl)	90 [80–107]
Bilirubin (mg/dl)	0.5 ± 0.1
Alanine aminotransferase (U/L)	14 [9 − 21]
Sodium (mmol/L)	138.6 ± 2.7
MMSE score	28 [27–29]
BDI-II score	
Normal	49 (71)
Mild	9 (13)
Moderate	9 (13)
Severe	2 (3)
CFF (Hz)	42.1 ± 1.1
PHES score	−1.5 ± 2.4

Note: Continuous variables given as mean ± standard deviation or median [interquartile range]; categorical variables as count (percentage). IQR: interquartile range; CKD: chronic kidney disease; MMSE: Mini-mental State Examination; BDI-II: Beck depression inventory; CFF: critical flicker frequency.

### Critical flicker frequency (CFF) and PHES

**Figure 1.**
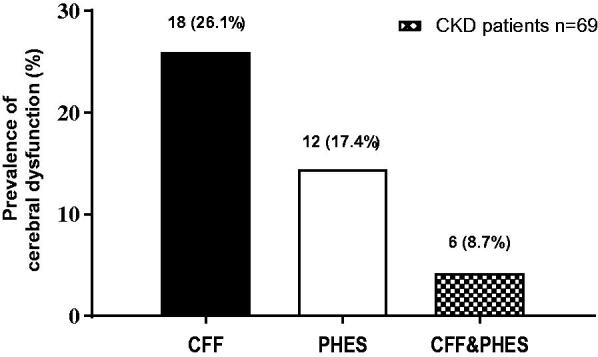
Prevalence of cerebral dysfunction. Note: [PHES: p 0.004, Flicker p = 0.002]. CKD: chronic kidney disease; PHES: Psychometric Hepatic Encephalopathy Score; CFF: critical flicker frequency.

The study participants (all with CKD) scored a mean CFF of 42.1 ± 1.1 Hz and a mean PHES score of −1.5 ± 2.4. The prevalence of cerebral dysfunction detected through PHES criteria (a score below 4) was 12 cases (17.4%), and the prevalence of cerebral dysfunction detected through CFF (≤39 Hz) was 18 cases (26.1%). Only six cases (8.7%) were diagnosed with this condition by both methods (Figure 1). Of the twelve cases detected by PHES, seven were on hemodialysis, five on peritoneal dialysis, and one patient in pre-dialysis (*p* = 0.5). Of the 18 cases with cerebral dysfunction by CFF, 13 cases were in hemodialysis and five cases in peritoneal dialysis (*p* = 0.2).

### Global cognitive function and depression

The participants scored an average of 28 points on the MMSE (out of a maximum of 30). Cognitive impairment (scores ≤24) was detected in seven patients: five of them were on hemodialysis and two on peritoneal dialysis.

**Figure 2.**
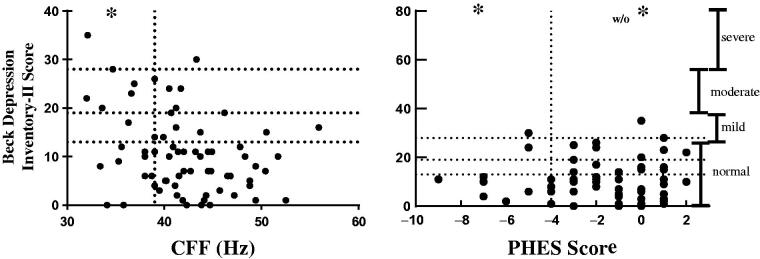
Correlation between PHES and CFF scores and severity of depression by Beck Depression Inventory. *Cerebral dysfunction; PHES: Psychometric Hepatic Encephalopathy Score; CFF: critical flicker frequency.

Twenty CKD patients (29%) had some grade of depression as defined by their score on the BDI-II. Severity of depression by Beck score was negatively correlated with CFF score (r= −0.28, *p* = 0.01), and was not correlated with PHES (*r* = 0.09, *p* = 0.45). Figure 2 shows levels of depression in our study participants.

### Retinopathy as confounding variable

As retinopathy can be an important confounding factor on the CFF, we compared 6 participants with diabetes and mild retinopathy against 33 participants with diabetes but without retinopathy. The mean CFF score was 37.4 ± 6.5 vs 42.5 ± 5.1 for those with and without retinopathy, respectively (*p* = 0.03). Of the six diabetic participants who had retinopathy, five of them had cerebral dysfunction as diagnosed by CFF. Of the 33 diabetic participants without retinopathy, only seven of them had cerebral dysfunction diagnosed by CFF (*p* = 0.007).

The mean PHES score was −3.2 ± 1.09 vs. −1.5 ± 2.5 for those with and without retinopathy, respectively (*p* = 0.1). One patient of each group (of the six with retinopathy and the 33 without retinopathy) did not finish the PHES test. Therefore, the proportion of diabetic participants with cerebral dysfunction diagnosed by PHES was 3 of the 5 diabetic participants with retinopathy and 6 of the 32 diabetic participants without retinopathy (*p* = 0.08).

CFF was closely related to retinopathy and depression; we analyzed the effect of both potential confounding factors and repeated the analysis excluding 8 of 69 patients with mild retinopathy or severe depression. The CFF mean of these 61 patients was 43.1 ± 4.4 Hz while the total study population mean was 42.1 ± 1.1 Hz.

### Changes in CFF scores pre and post hemodialysis

**Figure 3.**
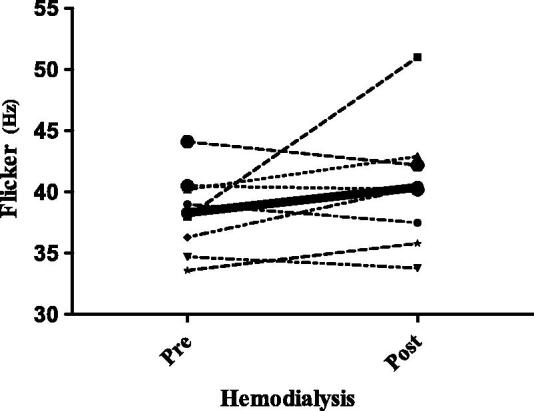
Changes in CFF pre- and post-hemodialysis.

As no clear guidelines exist on the ideal moment to administer the CFF in populations with kidney disease, the test was carried out both before and after hemodialysis in the first eight patients recruited. The goal of this pre- and post-dialysis testing was to determine if participants performed differently according to the test’s relationship to hemodialysis. The mean CFF score was 38 ± 3.4 Hz pre-hemodialysis vs. 41 ± 4.9 Hz post hemodialysis, respectively (*p* = 0.1). The mean KtV value was 1.8 ± 0.46. Because we observed that certain values declined while others improved after hemodialysis, we decided to apply the CFF at any time in the rest of the patients. Because those eight patients had 2 CFF measurements, we utilized the pre-dialysis value in the general analysis (these patients’ measurements are shown in Figure 3).

### Cognitive impairment

Five of 7 (71.4%) participants with cognitive impairment vs. 13 of 62 (20.0%) of those without cognitive impairment were diagnosed with cerebral dysfunction by CFF (*p* = 0.01). The mean CFF score was 37.9 ± 5.83 for those with cognitive impairment vs. 42.7 ± 4.9 for those without cognitive impairment (*p* = 0.02). Using PHES, 2 of 6 vs. 11 of 61 patients (*p* = 0.3), in those with and without cognitive impairment, respectively, were diagnosed with cerebral dysfunction. The mean PHES score was −2.17 ± 2.1 vs −1.49 ± 2.4, respectively (*p* = 0.5).

We analyzed the agreement between the MMSE, PHES, and CFF, finding that MMSE and CFF had an absolute agreement of 78.3% and a kappa coefficient (k) of 0.297 (*p* = 0.004). The MMSE and PHES had an absolute agreement of 77.6% with *k* = 0.100 (*p* = 0.3), and the CFF and PHES had an absolute agreement of 86%, *k* = 0.311 (*p* = 0.001).

## Discussion

This study is the first to describe the prevalence of cerebral dysfunction linked to UE – characterized by subtle neuropsychological and neurophysiological alterations without clinical alterations – diagnosed through CFF and PHES in stage 5 CKD patients. Up until the time of this manuscript’s publication, these two tools had only been applied to detect minimal hepatic encephalopathy in patients with cirrhosis. The prevalence of cerebral dysfunction was 26.1% as diagnosed by CFF and 17.4% by PHES. These tests do not appear to be greatly affected by the specific moment in which they are administered, as participants scored roughly equally before and after hemodialysis. The prevalence of neuropsychological and neurophysiological alterations was higher when detected by these methods that when using MMSE (10.1%), probably because both of them were designed to evaluate specific domains affected by minimal encephalopathy [33].

Although the pathophysiology of hepatic encephalopathy is different from uremic encephalopathy, both share the characteristic of presenting reduced cognitive functions in their early stages as a result of nonspecific cortical involvement. This similarity is why we propose evaluating both syndromes with the same instruments. Numerous tests exist to measure CHE in cirrhotic patients, with PHES and CFF enjoying widespread usage because they are easy to apply and have good external validity. It is important – for providers, patients, and relatives – to also identify similar cognitive disorders and neurological and physiological changes in CKD patients.

Kidney function and the type of associated replacement therapy are associated with cognitive impairment [34–37]. Previous studies have found that cognitive impairment can decline when patients start hemodialysis [38–40], and in our study, 61% of the patients were on hemodialysis. Of our seven patients with cognitive impairment, five were on hemodialysis and two on peritoneal dialysis, although these differences were not statistically significant. However, this appears to be a trend and should be investigated further. While several tools are available to assess cognitive impairment in renal patients, most are based on completing pencil and paper tasks and subject to biases. Factors such as age, education level, and occupation can affect these tests’ final results [10] but PHES and CFF are adjusted to these factors.

One strength of this study is that, in addition to administering PHES and CFF tests to our participants, we also evaluated cognitive impairment through an MMSE. We then performed a sub-group analysis to compare participants with and without cognitive impairment. MMSE value appeared to be associated with poorer CFF results but did not appear to be associated with PHES results. Because the MMSE, CFF, and PHES all ultimately evaluate cognitive impairment, one might expect a high degree of agreement between them. However, only PHES and CFF had a strong level of agreement, and MMSE had a moderate agreement with CFF (78%) and PHES (78%), respectively. This discordance could be because each test evaluates a distinct cerebral function; for example, CFF is hypothesized to be a measure of overall neural integrity and firing rate. The processing of CFF involves retinal and cortical processes and may be a unique predictor of executive dysfunction. Taking the PHES involves different cortical and subcortical areas. The PHES comprises different neuropsychological tests that assess diverse cognitive functions such as visual perception, construction, attention, psychomotor speed, cognitive flexibility, planning, and self-monitoring. As both PHES and CFF methods explore different brain functions, they could be complementary, rather than equivalent.

Conditions like depression could affect individuals’ performance on the CFF and PHES. We found a negative correlation between BDI-II score (with higher scores signifying more severe depression) and CFF score (lower scores signifying more severe encephalopathy). However, no correlation was found between BDI-II scores and PHES scores. These findings suggest that PHES could be better than CFF for detecting cerebral dysfunction in the presence of depression. Our study found that those on hemodialysis had a higher prevalence of depression and cognitive impairment, in line with previously reported findings [37,41].

In Mexico, the primary cause of CKD is diabetes, which is why we carefully evaluated diabetic retinopathy’s role in limiting the usage of CFF and PHES. As 50% of our study participants had diabetes, we compared the results of those with and without a diagnosis of diabetic retinopathy. In this study, six patients had mild retinopathy (moderate and severe retinopathy were excluded) that did not seem to represent a physical limitation. However, the result of CFF was modified by retinopathy, while PHES was not. In this sense, PHES could be a better tool for people with diabetes, as CFF depends on visual capacity.

As with all cross-sectional studies, this study suffers from certain limitations. The primary limitation is that the methodology did not allow us to draw conclusions about causality, but rather provides associations and inspirations for further hypotheses and lines of work. Additionally, as all patients were recruited from a hospital setting, their clinical and socioeconomic characteristics may differ from those in the general Mexican population. Moreover, as this study was only conducted in a single hospital, the results may not be generalizable to the full population. Finally, there are no additional diagnostic methods – such as an electroencephalogram (EEG) or imaging to identify structural changes in the brain [42] – that could serve as a ‘gold standard’ against which to compare the tests proposed here. We decided to use the MMSE because of it is a cognitive test that is widely used in clinical practice and because patients’ performance on the MMSE is often compared to their results on a PHES and CFF. We are aware there are other screening tests that have more items related with attention and executive function, like the MoCA. It is probable that we would have found more abnormalities if we would had selected any of these more other tests to assess cognitive function. Future studies should, therefore, use these more comprehensive screening tests that have higher sensitivity and specificity to detect mild cognitive impairment.

Previous research using CFF and PHES to measure minimal hepatic encephalopathy has revealed that people with this condition suffer changes in memory, concentration, and perceptions. These changes may be so subtle that they are only perceived by those close to the patient and never even reported to a clinician. However, these subtle changes have a very real impact on aspects of life and may lead to developing overt encephalopathy.

## Conclusion

This study has revealed a high prevalence of cerebral dysfunction among individuals with chronic kidney disease, albeit without an established diagnostic test. It is essential to identify cerebral dysfunction when is in early subclinical stages to reduce preventable negative outcomes, such as traffic and work accidents. We posit that PHES and CFF can be used together for diagnosing cerebral dysfunction in populations with kidney disease and call for further exploration of this diagnostic process.
